# Effect of Beverage Composition on Radio Frequency Identification (RFID) Performance Using Polyethylene Terephthalate (PET) Bottles for Smart Food Packaging Applications

**DOI:** 10.3390/foods13050643

**Published:** 2024-02-20

**Authors:** Ethan Claucherty, Danielle Cummins, Angelica Rossi, Bahar Aliakbarian

**Affiliations:** 1 The Axia Institute, Michigan State University, 1910 W. St. Andrews Rd., Midland, MI 48640, USA; clauche9@msu.edu (E.C.); cummi337@msu.edu (D.C.); 2 Department of Civil, Chemical, and Environmental Engineering, University of Genoa, 16145 Genoa, Italy; angelica.rossi0397@gmail.com; 3 Department of Biosystems and Agricultural Engineering, Michigan State University, 524 S Shaw Lane, East Lansing, MI 48824, USA

**Keywords:** radio frequency identification (RFID), tag performance, food packaging, food waste, food traceability

## Abstract

Radio frequency identification (RFID) technology is crucial in revolutionizing the food supply chain and combating global food waste. However, this technology faces challenges in full integration due to disruptive effects on tags caused by the dielectric properties of food and beverage ingredients, chemical constituents, and their packaging. This paper aims to demonstrate the effect of packaging and beverage contents on RFID tag performance. Three commercially available ultra-high frequency (UHF) RFID tags with different designs were tested on polyethylene terephthalate (PET) bottles, measuring tag performance through sensitivity, backscatter, and read range in the presence of various water-based solutions and commercially available beverages. The results highlight the substantial impact of the beverage type and tag design on RFID performance. The results of this study showed that tag 3 was the most consistent and readable tag amongst those tested in the presence of different beverage contents. Tag 3 resulted in a sensitivity ranging from −0.49 to −2.01 dBm, backscatter from −38.16 to 43.59 dBm, and read range from 1.58 to 1.88 m, while tag 1 performed the best in the presence of an empty PET bottle resulting in a sensitivity of −20.78 dBm, backscatter of −23.65 dBm, and read range of 16.34 m. The results of this study can be used for further investigations to develop a mathematical model that predicts the RFID tag performance based on the food composition. This model will be helpful for the design of the tags while facilitating the adoption of smart packaging for food traceability.

## 1. Introduction

For years, traditional packaging has been adequately delivering food products to customers in acceptable condition by protecting and preserving the item. Nevertheless, the last two decades have presented several social and logistical changes [1] that have had a significant impact on supply chains, which have also altered food supply chains. These disruptions, like the COVID-19 pandemic [2], have altered distribution in food supply chains, requiring a push for novel developments in advanced packaging systems [3,4,5]. Food safety is a primary player in public health. Regulations regarding the quality of food protect consumers from any variety of foodborne illnesses and unforeseen complications from cross-contamination. Food waste is another important issue that has been plaguing food supply chains [6,7,8,9]. It has been shown that RFID-based smart packaging can achieve effective management of perishable food by sensing changes in biomarkers for freshness assessment, thereby reducing food waste [10]. Historically, packaging materials were designed to prevent food degradation, contamination, and function as a barrier to chemical and mechanical stresses [11]. Food packaging has evolved from being a passive barrier to having a more active role in preservation and traceability along the supply chain [5,12,13]. Food companies look to strengthen their supply chain management and logistics to guarantee the highest level of control. Achieving this can improve track and trace efficiency, leading to enhanced quality, safety, and cost/benefit objectives. Affordable, innovative systems and technologies have been developed that enable the generation and storage of data at an item or batch level, allowing for increased visibility and insight throughout the supply chain. In order for this to happen, a supply chain must prioritize item-level identification for this level of granularity to be achieved. This is only possible if each item is provided with a unique identity that is easily and efficiently recognized through the entire supply chain [4,14,15,16].

Radio frequency identification (RFID) systems optimized with real-time monitoring technologies have already been adopted for traceability purposes in many supply chains including apparel, electronics, pharmaceuticals, and food [17]. In these use cases, Ultra-high frequency (UHF) RFID is most popular. This technology’s ability to print an integrated circuit (IC), and its competitive ability to read longer distances makes it the primary choice when working to improve supply chain visibility [18]. However, to aid the adoption of this technology, several challenges must be overcome. Several factors strongly influence the energy harvesting capability of RFID tags including material, shape, composition, packaging size, and even the contents inside the packaging. One issue in applying UHF RFID at the item level is the system’s inability to operate efficiently when in close proximity to different kinds of substrate materials [19,20,21,22]. Another challenge is the cost associated with implementation [23]. Although the introduction of RFID in the agri-food supply chain increases its productivity, not all companies will decide to use it due to its additional cost [14,23]. Furthermore, integrating a UHF RFID-based traceability system in the food and beverage industries represents a challenge not only due to the composition of external packaging, but also the high water content in food which notably impacts RF performance. Materials that are high in lipid concentration are less critical than food products containing a large quantity of water [20,21,22,24]. RFID tag readability, when in presence of an aqueous solution, is a function of four key parameters which include the chemical compound, concentration of the compound in the aqueous solution, temperature of the solution, and orientation of the tag in respect to the UHF RFID reader antenna [19].

A better knowledge and understanding of the limitations of UHF RFID systems when identifying the appropriate packaging material for different food products will help avoid failure in implementing the RFID-enabled tracking system [24]. Only a few experimental works have been conducted to assess the effect of different liquids in the vicinity of RFID tags attached to bottles [18,22,25,26,27]. Xi et al. 2012 [28] found that one way to solve this problem is to avoid the liquid by placing the tag on the neck of the bottle, but positioning the tag here only works when the bottle is standing upright. Gonçalves et al. 2014 [29], proposed solution was to embed the tag inside a cork, but due to the complicated design, the fabrication process would be expensive. Other efforts for performance improvements have focused on changing the RFID tag antenna design by considering the effects on RFID readability when attached to bottles filled with liquid with different dielectric properties [20,21,22,30]. While previous studies have explored the influence of aqueous solutions on RFID performance, there remains a significant gap in understanding how food products specifically affect the read range of UHF RFID-enabled packaging. This research addresses this notable gap in knowledge. Given the potential advantages of real-time information gathered from RFID-enabled packaging, it becomes imperative to delve into the unique impact that various food materials can have on RFID performance. This study seeks to contribute to the existing body of knowledge by shedding light on the novel aspect of how food products interact with UHF RFID technology, enhancing our understanding of their influence on the performance of RFID-enabled packaging.

This work aimed to assess the performance of different commercially available UHF RFID tags with assorted designs when they are attached to polyethylene terephthalate (PET) containing different solutions. This packaging material was selected because of its frequent applications in the food and beverage sector. The liquids used were different water-based solutions containing a specific concentration of Sucrose and Citric Acid to mimic the composition of commercially available orange and apple fruit juices. This study builds upon previous research conducted by Rossi et al. [26], which aimed to identify the optimal labeling position for PET and aims to assess any correlation between the material composition of food and the resulting losses in RFID tag performance. The result of this study can be used to effectively design UHF RFID tags that can be used for tracking of the beverages contained in PET bottles.

## 2. Materials and Methods

### 2.1. Materials, Reagents, and Solutions

**Figure 1:**
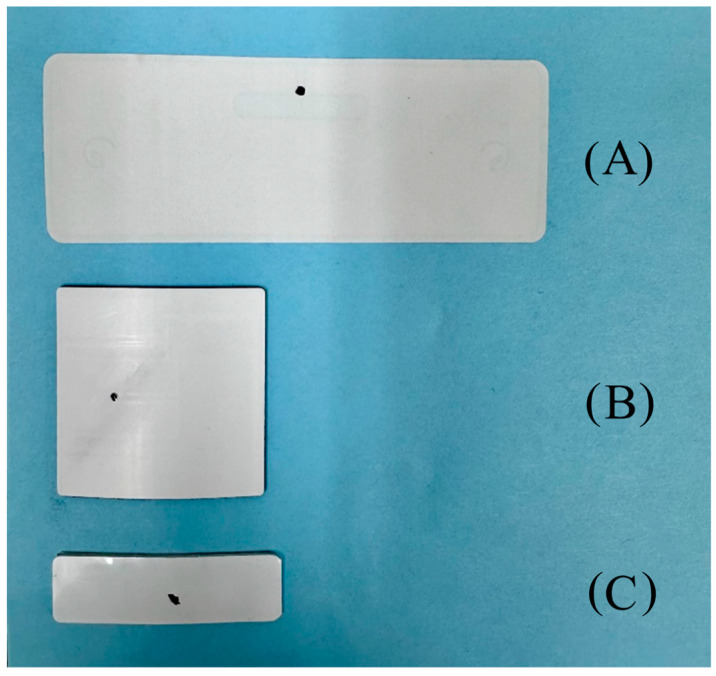
Three different commercially available UHF RFID tags used in this work from top to bottom (**A**) tag 1, (**B**) tag 2, and (**C**) tag 3. The black dot illustrates the integrated circuit (IC).

Three commercially available UHF RFID tags were used for testing. These tags were bought from AtlasRFIDstore.com accessed on 14 November 2023 and are indicated as tag 1, tag 2, and tag 3. The tags used are shown in Figure 1 below.

**Figure 2:**
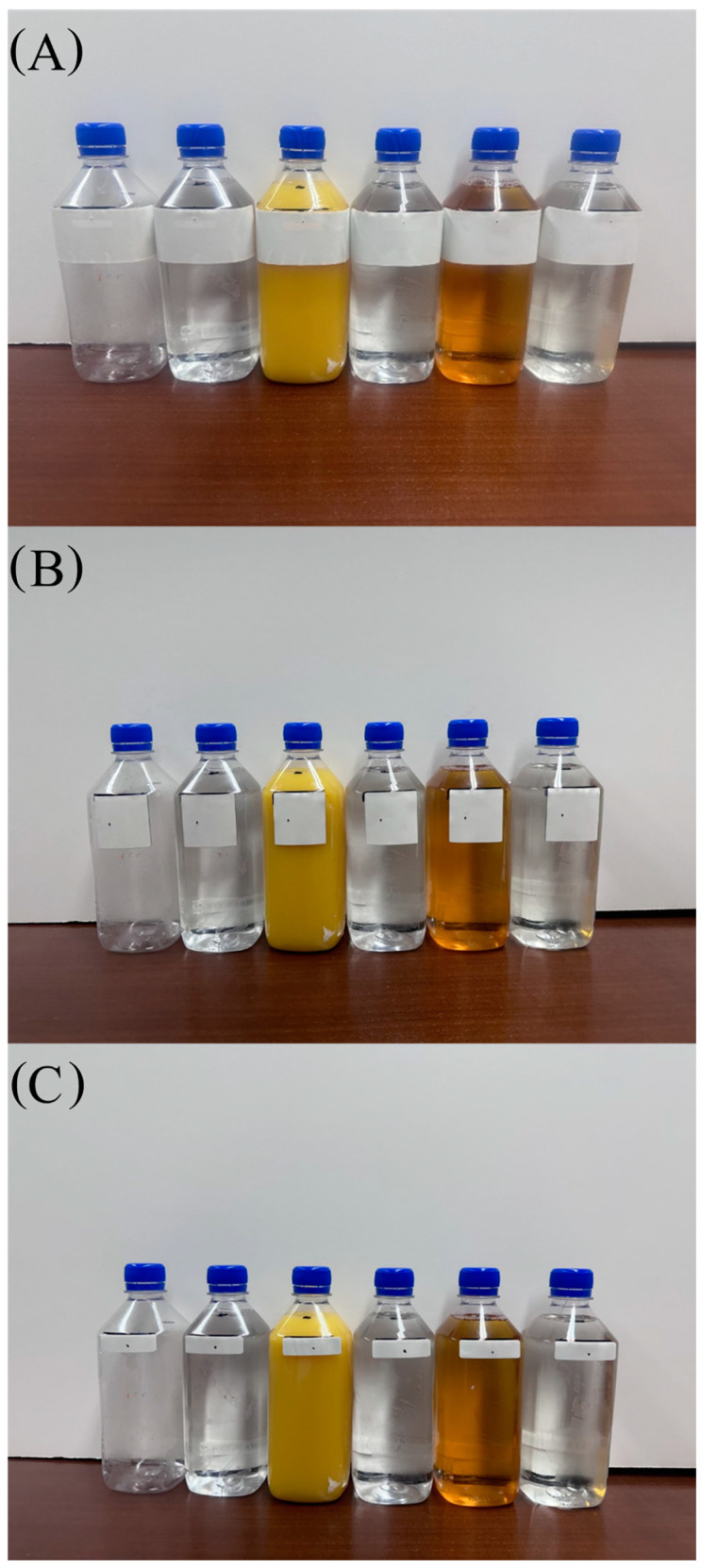
Commercial PET packaging filled with five different solutions + empty PET container for each tag evaluated. (**A**) tag 1, (**B**) tag 2, (**C**) tag 3.

The packaging type used in this study were bottles made of polyethylene terephthalate (PET), which is used for a variety of beverages in the food industry. These bottles were purchased at a grocery store and are shown in Figure 2. The bottles were emptied, washed, and dried before testing.

Commercially available orange juice and apple juice was used to investigate the effects of beverages on RFID tag performance. These products were bought at the grocery store, refrigerated prior to analysis, and allowed to come to room temperature prior to testing. The nutrition facts of each beverage were stated on their labels and complied with Nutrition Labeling and Education Act (NLEA) of 1990. We selected PET as a significant packaging type for beverages, with a specific emphasis on apple and orange juice. These juices were chosen to represent beverages containing Sucrose and Citric Acid, as these two factors can impact the dielectric properties of the product, consequently affecting RFID performance. The beverages (orange juice and apple juice) were chosen to explore potential worst-case scenarios for RFID performance in food packaging. This choice was made because these beverages represent products containing water, sugar, and acid, each with a different color and soluble solid percentages. Regarding colloidal systems and chemical composition: orange juice is highly acidic and contains more dispersed solids, while apple juice has a high Sucrose content and is a clear liquid.

The following water-based solutions were prepared to assess the effects of food contents on RF performance. Citric Acid and Sucrose, both highly pure (>98%) chemical compounds, were purchased from Sigma Aldrich, St. Louis, MO USA. The aqueous solutions were prepared with analytical grade compounds in deionized water to achieve a 1% Citric Acid and 12% Sucrose solution to mimic the average reported concentrations of three different commercially available beverages of orange juice and four commercially available apple juice beverages, shown in Table 1 and Table 2. The three commercially available orange juice brands had Citric Acid contents reported by Penniston et al. [31] and Weikle [32], seen in Table 3. The sugar % per serving and Citric Acid % per serving were calculated using the following two equations.

(1) Total Sugars gServing Size oz∗(28.35 goz)=% Sugar by Volume

(2) CA Concentrationmgoz∗1 g1000 mgServing Size oz∗28.35 goz=% Citric Acid by Volume

### 2.2. RFID Performance Testing Methodology and Parameters

**Figure 3:**
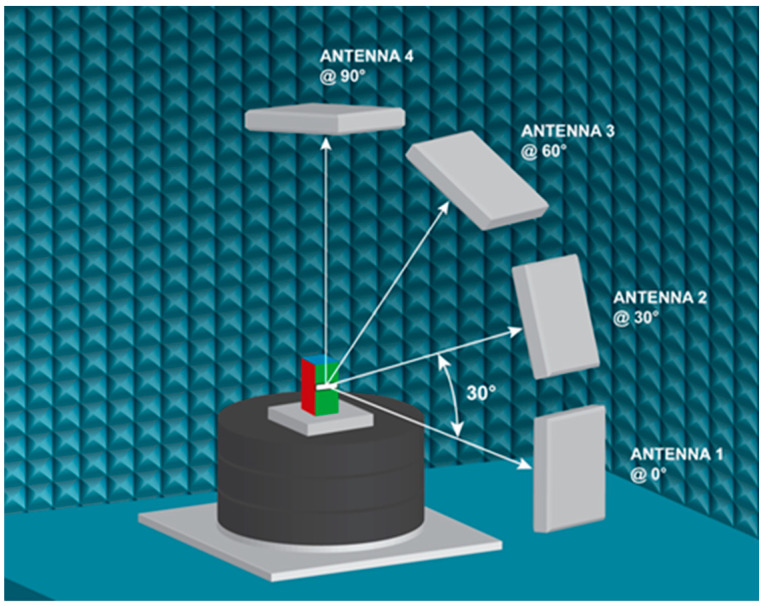
Illustration of anechoic chamber setup used for RFID performance testing.

This research aims to identify how the performance of various commercially available UHF RFID tags, featuring different designs, changes when attached to polyethylene terephthalate (PET) packaging containing diverse solutions. To determine the impact of material property on RFID performance, all RFID performance measurements were conducted using a C50 Voyantic^®^ (Helsinki, Finland) chamber used in conjunction with the Voyantic Tagformance^®^ measurement system. The C50 Voyantic^®^ chamber is an anechoic chamber that has a 50 cm nominal measurement distance and the circular arrangement of four antennas each mounted at 0°, 30°, 60°, and 90° with respect to the test platform. The antennas are mounted so that their horizontal polarization plane is parallel to the floor of the anechoic chamber. The test platform rotates 0° to 360° with a minimum of 1° increments (Figure 3).

To assess the influence of the substrate on the RFID tag, the samples were filled with 500 mL ± 5 mL of water-based solutions or beverages and fixed with each type of RFID tag at the top of the container. This position of RFID on the PET bottle was optimized in the previous research by Rossi et al. [26]. Empty bottles tagged in the same way were used as control samples. One at a time, each sample was placed at the center of the rotating table of the anechoic chamber, with the UHF RFID tag’s integrated circuit (IC) fixed at a focal point 50 cm away from the antenna array facing antenna 1. Tests were performed at room temperature between 20 and 22 °C and relative humidity between 24% and 36% as instructed by the equipment manufacturer. To evaluate the tag performance, two separate tests were completed with Voyantic^®^ software (version 13.5). The parameters selected to analyze tag performance were Power on tag Forward (PoTF) or sensitivity, Power on tag Reverse (PoTR) or backscatter and the Theoretical Read Range Forward (TRRF) or theoretical read range. The two tests executed to assess the overall RFID tag performance were threshold sweep, which shows how much energy is needed to stimulate a response from the tag (PoTF) and how much power is reflected back after an effective response from the tag (PoTR) and orientation sweep, which displays the radiation pattern and describes how well the tag is readable from different angles.

The first set of tests were aimed to determine the performance of the various RFID tags on PET and solutions/beverages throughout the entire UHF band (800–1000 MHz) in which these antennas operate. This is 866–869 MHz in Europe, 902–928 MHz in America, and 950–956 MHz in Asia [33], with communication for these devices occurring in the far-field region. Each beverage/solution and tag were assessed once. A second threshold sweep was then performed with the angle rotation fixed at 0° and operation frequency of 915 MHz, which is the central frequency for the Federal Communications Commission (FCC) approved North America UHF regulation range. Each beverage/solution and tag were then assessed thirty times. Following the threshold sweep, an orientation sweep was performed at 915 MHz in 10° rotations for an in-depth look at the radiation pattern [33]. The test on each beverage/solution and each tag was repeated five times. This research followed a similar procedure to the one performed by Expósito and Cuiñas [34], where the authors tested several different RFID tags in the presence of white wine to assess their performance.

### 2.3. Statistical Analyses

All data analyses were performed using Microsoft Excel with the Data Analysis Toolpak (version 2312, Redmond, WA, USA). Results were represented as the mean ± standard deviation (SD). Statistical differences between two or more groups were analyzed using one-way analysis of variance (ANOVA) followed by a post hoc student’s *t*-test with *p* < 0.95 [35]. Statistical significances are explained in the text and depicted in Tables and Figures using different letters.

## 3. Results

### 3.1. Effect of Aqueous Solutions and Beverages on Tag Sensitivity and Backscatter

**Figure 4:**
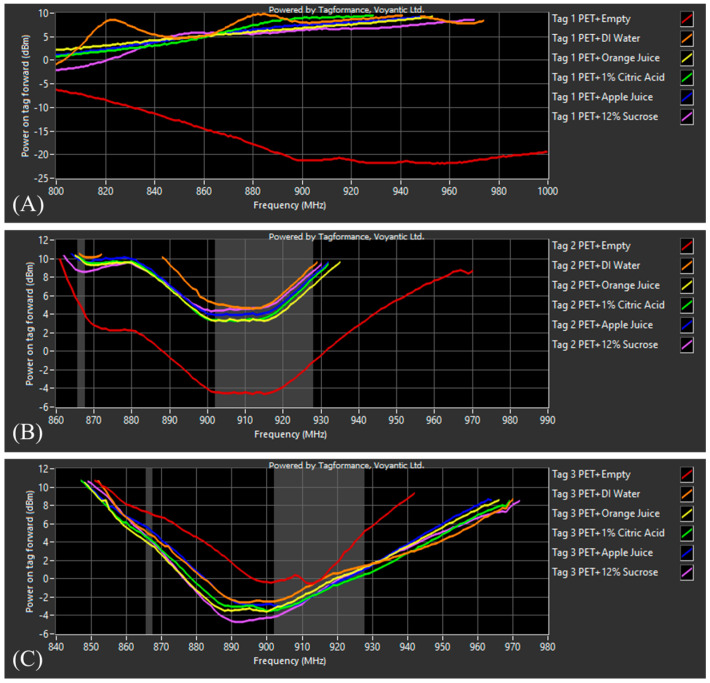
Power on Tag Forward (PoTF) results from 800–1000 MHz for (**A**) tag 1 on PET + six test solutions, (**B**) tag 2 + six test solutions, and (**C**) tag 3 + six test solutions.

A preliminary threshold sweep was performed using the three tags and PET containers filled with the different solutions/beverages to identify the correct tag orientation in respect to the 0° antenna in the Voyantic^®^ chamber. The results have shown that all three tags were most readable when horizontally attached to the substrate. Thus, all labels were attached to the bottles horizontally. RFID labeled bottles with or without solutions/beverages were placed in the chamber one at a time and measured through the frequency range of 800–1000 MHz. Figure 4 displays the PoTF or tag sensitivity results of all three tags on the PET substrate with the different controls and test solutions.

The preliminary results show that tag 1 was slightly more tuned for the European Telecommunications Standards Institute (ETSI) approved European UHF regulation range of 866–869 MHz with the empty PET bottle drastically outperforming all the other solutions and beverages. Tag 2 was more tuned to the FCC range of 902–928 MHz, with the empty PET bottle also outperforming the other solutions and beverages. Tag 3 was more tuned to the FCC range; however, the empty PET bottle performed the worst when compared to the other solutions. Across all three tags, the results show little variation in sensitivity between solutions; however, to confirm these results, a second threshold sweep was performed in the middle of the FCC range at 915 MHz using thirty samples of each tagged solution and beverage to provide more statistically robust analysis.

The results shown in Table 4 and Table 5 depict the PoTF (sensitivity) and PoTR (backscatter) of each tag on PET with the different test solutions and confirms the preliminary threshold results. The performance of tag 1 surpassed that of all other tags and solutions tested. When tag 1 was attached to the empty PET bottle a sensitivity of −20.78 ± 0.08 dBm and a backscatter of −23.65 ± 0.13 dBm were achieved. While the sensitivity was the best using an empty bottle, the tag performance changed significantly when the bottle was filled with different solutions. When the bottle was filled, among all solutions using tag 1, 1% Citric Acid resulted in the worst sensitivity (9.67 ± 0.12 dBm) and backscatter (−37.54 ± 0.32 dBm), and 12% Sucrose resulted in the best sensitivity of (7.00 ± 0.34 dBm), and backscatter of (−35.34 ± 0.36 dBm). This confirms the research hypothesis that the product formulation has an impact on tag performance and should be considered during design and implementation.

Tag 2 and tag 3 were also successfully read in the presence of all solutions, with tag 2 producing the second highest performance when used on an empty PET bottle. This resulted in a sensitivity of −4.88 ± 0.11 dBm and backscatter of −37.32 ± 0.43 dBm. Tag 3 was the best performing tag on the PET bottle filled with different solutions. A similar trend to tag 1 was also noticed when the bottle was filled with different solutions, resulting in a reduced performance compared to the empty bottle. Using tag 3, the highest performance was seen on PET with apple juice with a sensitivity of −2.01 ± 0.19 dBm and backscatter of −42.04 ± 0.26 dBm. The worst performing solution was DI water with a sensitivity of −0.49 ± 0.05 dBm and backscatter of −43.59 ± 0.23 dBm. Tag 3 has proven to be the most consistent, exhibiting the least sensitivity to material composition when compared to the other two tags. A statistical analysis using a one-way ANOVA and post hoc student’s *t*-test (*p* < 0.05) was performed to determine the effect of tag design on sensitivity and backscatter for each solution. The results demonstrating statistical differences (*p* < 0.05) with different letters (a to c) among the three tags are presented in each row of Table 4 and Table 5. In 2023, Rossi et al. [26] used the same tags and PET containers and their findings were consistent with what was found in the present study in that when these tags were placed at the top of the empty containers, the results were very similar [26]. This indicates that tag design, placement, and packaging content are all critical in RFID tag performance. Other researchers have performed similar testing using different test solutions often encountered in food products (salt, organic acids, sugars, and alcohol) in bottles made of HDPE tagged with different UHF RFID tags [36]. The results from this study showed that a high NaCl content, like that seen in salty liquids and brine, and dissolved week acid molecules strongly impaired RFID functioning, especially at room temperature, while the presence of other organic compounds dissolved in water (Sucrose, Ethanol) did not have a strong effect on tag readability at the considered concentration [22].

Overall, based on the results presented in this section, we can conclude that tag performance varies when the container is filled with a solution. Furthermore, we observed statistical differences (*p* < 0.05) in tag performance when different tag designs were used. Therefore, material composition should be given strong consideration when designing an RFID tag.

### 3.2. Effect of Aqueous Solutions and Beverages on Theoretical Read Range

Theoretical read range is one of the most essential parameters for a RFID tag, which is the maximum interrogation distance between the reader and the tag [37]. Theoretical read range is a critical factor demonstrating how effectively a tag can be read by a reader at varying distances. This parameter is of critical importance for industrial applications where precise and reliable data acquisition over varying distances is essential for optimizing operational processes and ensuring the efficiency of RFID technology in diverse industrial settings. Other researchers have investigated the read range using different tags on a variety of plastic and glass containers filled with beverages such as water, soda, cola, mango juice, cooking oil, beer, and wine [18]. The results showed the largest read range was observed from front direction on a water bottle, whereas the lowest read range was observed on the soda beverage which may be due to the high sodium bicarbonate content [8].

**Figure 5:**
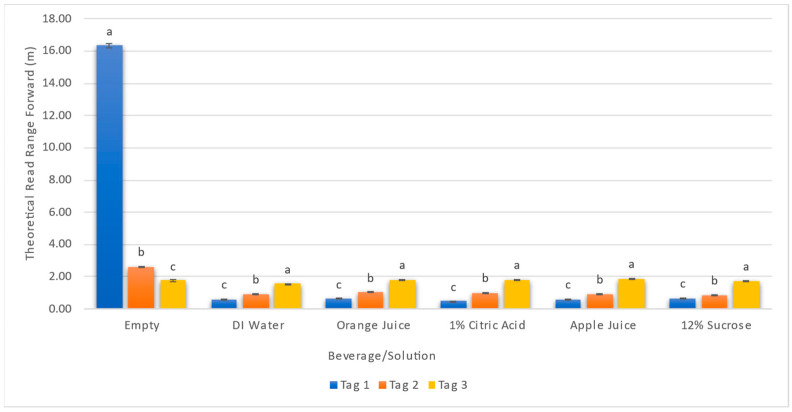
Theoretical read range forward of three different tags on five different solutions and an empty PET control. Different letters (from a to c) within a beverage/solution indicate significant difference (*p* < 0.05) between each tag.

Three tags on PET bottles filled with different beverages and aqueous solutions were assessed thirty times, and the results are presented in Figure 5. The results show that the furthest theoretical read range was observed using tag 1 on an empty PET bottle with a read range of 16.34 ± 0.16 m followed by tag 2 on the empty PET bottle with a read range of 2.62 ± 0.03 m. Tag 3 had lowest readability for an empty PET bottle with a read range of 1.76 ± 0.04 m. These results are consistent with the results seen in Section 3.1 confirming the correlation between read range, tag sensitivity and backscatter. When tag 1 was used on a PET bottle filled with solutions, the read range increased 36.7% between its worst performing solution of 1% Citric Acid (0.49 ± 0.01 m) and its best performing solution of 12% Sucrose (0.67 ± 0.03 m). Tag 2 followed a similar trend, increasing 19.5% between 12% Sucrose (0.87 ± 0.01 m) and orange juice (1.04 ± 0.02 m). Tag 3 read ranges only had a difference of ~18.9% between its worst solution of DI water (1.58 ± 0.01 m) and its best solution, apple juice (1.88 ± 0.04 m). Although tag 3 had the lowest read range for an empty PET bottle, it also had the most uniform and consistent read ranges between all test variables when compared to tag 1 and 2. Statistical differences (*p* < 0.05) among different tags have been shown with different letters (a to c) in Figure 5. Similarly to the results of sensitivity and backscatter, the tags exhibited varying performances (*p* < 0.05) for each solution, confirming the significance of material composition and the selection of tags for different use cases.

### 3.3. Effect of Aqueous Solutions and Beverages on Radiation Pattern

**Figure 6:**
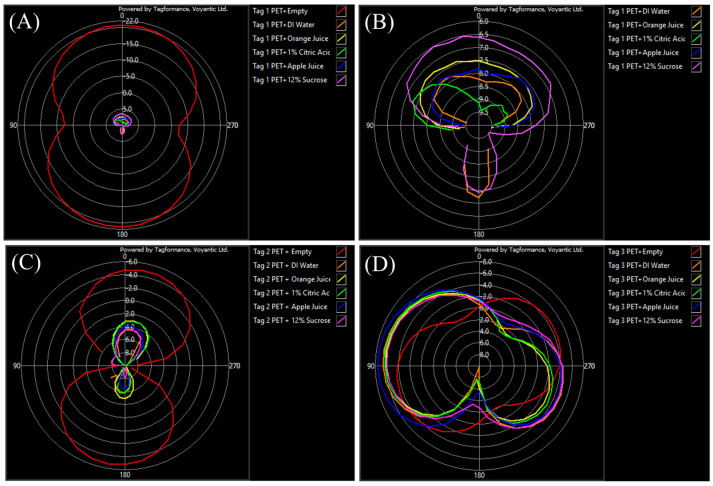
Orientation pattern for (**A**) tag 1 with all six test items, (**B**) tag 1 results without the empty bottle, (**C**) Tag 2 with all six solutions, and (**D**) tag 3 with all six solutions.

An orientation sweep is a critical performance parameter that is usually included on an RFID tag data sheet and is used to visually show the radiation pattern of an antenna. This can give an insight into how a tag is affected by the item as well as the contents it is attached to when rotated 360°. The empty PET bottle is the highest performing tagged item in both tag 1 and 2, and this is verified by its orientation sweep with each tag, which can be seen in Figure 6.

The omnidirectional pattern created by the empty bottle exemplifies the most desired and uninhibited radiation pattern. It is the only test for tag 1 that did not have a dead zone, and similarly has the smallest dead zone in tag 2. This is most likely due to the lack of additional content composition components with different dielectric properties present inside the bottles. The other shapes and disfigurations in the graphs of the other substances are due to the presence of liquids and the effects of their additional contents. The results seen in Table 6 present the best and worst angles, as well as any dead zones which varied depending on the tag used and solutions inside the bottle. Tag 1 showed that the most frequent best performing angle out of all six test solutions tested was 180° and was observed with the empty bottle and DI water, while the worst angle of 260° was seen with the empty bottle, orange juice, Citric Acid, and apple juice. Tag 1 also had a large number of dead zones that ranged between 90–270°. In the tag 2 results, the most prevalent best angle was 350° and was observed with the DI water, orange juice, apple juice and Sucrose, while the most common worst angle of 300° was seen in DI water and apple juice. This tag had dead zones with all the tested solutions and ranged from 40 to 290°. Tag 3 showed the most consistency amongst the items tested with 80° appearing as the best angle in five of the solutions and seen with all minus the empty bottle and the most prevalent worst angle being 170° and seen in all besides the empty bottle and apple juice, which came close at 180°. This tag had no dead zones and was readable at all angles with all solutions.

## 4. Discussion

This research aimed to uncover, for the first time, the dynamic shifts in the performance of a variety of commercially available UHF RFID tags, each characterized by distinct designs. The critical performance characteristics, encompassing sensitivity, backscatter, theoretical read range, and orientation pattern, were thoroughly examined when these tags were applied to polyethylene terephthalate (PET) packaging containing a diverse array of solutions. The results demonstrated that each tag was affected by the presence of aqueous solutions with slight changes in the performance based on the composition of each solution. It was also shown that each tag exhibits distinct performance characteristics (*p* < 0.05) when attached to an empty PET bottle. Different tags required different quantities of power, sensitivity, to be successfully activated and read as a function of the substrate material they were attached to. The variance in tag performance due to tag design was further substantiated by the statistical analysis (*p* < 0.05). This is a crucial factor that needs to be considered for the wide adoption of RFID-enabled packaging for food applications.

Filling the bottles with different aqueous solutions caused a pronounced general worsening effect on tag readability in tag 1 and 2. Contrary to this, tag 3 demonstrated remarkable consistency, displaying minimal sensitivity to material composition when compared to the other two tags. Tag 1 performed the best in the presence of an empty PET bottle with a sensitivity of −20.78 ± 0.08 dBm, backscatter of −23.65 ± 0.13 dBm, and read range of 16.34 ± 0.16 m followed by tag 2 with a sensitivity of −4.88 ± 0.11 dBm, backscatter of -37.32 dBm, and read range of 2.62 ± 0.03 m. Tag 3 was found to be the best tag among those tested as it had similar performance in terms of sensitivity, backscatter, and read range across all of the tested solutions with the lowest variation among different solutions (*p* < 0.05). The results showed that tag 3 had a sensitivity ranging from −0.49 ± 0.05 to −2.01 ± 0.19 dBm, backscatter from −38.16 ± 1.55 to 43.59 ± 0.23 dBm, and read range from 1.58 ± 0.01 to 1.88 ± 0.04 m. The orientation pattern confirmed this by showing that the best and worst angles were nearly identical across all the solutions, with the best angle being 80° for all the solution filled bottles. The worst angle was identified at 170° for all solutions with the exception of 180° in the case of the apple juice filled bottle. Additionally, tag 3 was also the only tag that had no dead zones or areas where the tag could not be read as it rotated 360°.

Based on the antenna performance information on the data sheets provided by the different tag manufacturers, tag 1 was not designed to work in the presence of metals, while tag 2 and tag 3 were chosen because of their specific ability to be used on additional beverage packaging materials. Using RFID tags that can work on multiple substrates, including metal, is important not only because of the increasing use of metal nanoparticles (MNPs) which are currently being used in food packaging to protect, preserve and extend the shelf life of food [38], but also due to the presence of different equipment and process lines made of metal in the manufacturing facilities. The obtained results were found to be in accordance with the limited research found in the academic literature on this topic and, the effect water and aqueous solutions have on RFID tag performance. Among these, a few researchers have investigated the effect food products have on the performance of RFID tags using commercially available UHF RFID tags [36,39], while others have designed sensors for specific applications [34,40]. Barge et al. (2017) [36] investigated the effect of temperature and tag position on UHF RFID tag readability for beverage packaging. When labeling an empty flask, the power needed to be successfully activated and read the tag was extremely low. The authors concluded that improper tag positioning can worsen readability [36], which could lead to a tag being undetectable. The effect of chemical compounds on readability was assessed as well. Barge et al. (2019) [39] reported that the RFID tag reading range is highly influenced by tag orientation with respect to the antenna, as well as by the food product chemical composition. In the mentioned work, the effect of the food product temperature was also investigated. Expósito et al. (2011) [34] assessed the performance of different tag models attached to wine bottles. They realized a large measurement campaign which resulted in a general worsening performance effect in presence of wine. Liu et al. (2018) [40] designed a specific UHF RFID tag for liquid products in glass bottles. They investigated the reduction in RFID readability due to the presence of liquid. The reading ranges of the proposed tag were measured both for the empty and filled glass bottle. In their study, six liquid products were tested, and all caused a reduction in the reading range in a restricted range. Another cheap and compact UHF RFID tag that is stable in the presence of liquid was proposed in Björninen et al. (2011) [41], which developed a low-profile conformal RFID tag antenna specific for water bottle applications.

It is important to note that this manuscript focused on examining juices as processed products, and the authors understand the variation in raw materials’ origins and varieties. While we acknowledge that the information about the impact of RFID-enabled packaging on reducing food waste may not be entirely convincing, recent advancements in RFID technology show promise in addressing this concern. RFID can potentially play a crucial role in real-time monitoring and traceability, allowing for better inventory management and minimizing the risk of food spoilage. Various researchers have explored the potential prospects and challenges of RFID [10,16]. Their findings reveal a delay in the commercialization of food sensor technologies in packaging, attributed to limitations in research, constrained energy harvesting, RFID read range, and cost issues. Despite these drawbacks, there is anticipated growth in the coming years. This growth is driven by the demonstrated benefits of RFID sensing in other supply chains including retail. It is essential to acknowledge that the application of RFID for food waste reduction is a dynamic field, and we believe that this research has the potential to uncover additional avenues for exploration in the domain.

## 5. Conclusions

RFID serves as an enabler of smart packaging and its ability to furnish real-time information about a product and its journey from the farm to the fork is essential for ensuring product safety and authenticity. When combined with sensors like those for temperature detection, RFID-enabled packaging can help identify if a product has spoiled due to exposure to harmful temperatures. This information could help managers make corrective and preventative actions, thus reducing food waste. While previous studies have touched upon the impact of aqueous solutions on RFID performance, a crucial gap persists in comprehending how food products specifically influence the tag performance including sensitivity, backscatter, and read range of UHF RFID-enabled packaging. This study contributes to the existing body of knowledge by unveiling a novel aspect—how food products interact with UHF RFID technology, thereby enhancing our understanding of their influence on the performance of RFID-enabled packaging. In this study, three different commercially available UHF RFID tags were used on PET bottles filled with simulated apple and orange juice compositions as well as the equivalent commercially available juices. The major performance parameters including tag sensitivity, read range, and orientation patterns were determined. We focused on the PET as one of the major types of packaging used for beverages. We also focused on apple and orange juice to represent beverages with Sucrose and Citric Acid, two factors that can affect the dielectric property of the product, thus influencing RFID performance. The results underscore the importance of tailoring RFID tag designs to specific applications and highlight the significant influence of material on RFID tag performance. Although tag 3 was discovered to be the best among those tested, it does not represent the appropriate tag to be universally utilized for different packaging filled with different beverages. While this study does not identify a definitive “best-performing” tag for food packaging, it lays the groundwork for future exploration by RFID tag manufacturers and packaging experts. The absence of a universal RFID tag poses a substantial technical challenge in food supply chain applications. Future research, encompassing a larger sample size and diverse materials, is crucial to developing robust prediction models. This endeavor will not only refine RFID tag design for food and beverages but also assume a pivotal role in bolstering food safety, reducing food waste, and optimizing the efficiency of supply chains. This impact is particularly significant in critical sectors such as food and pharmaceuticals.

## Figures and Tables

**Table 1:** Beverage nutritional facts reported by label for four commercially available orange juice brands.

Orange Juice Brands	Serving Size (mL)	Total Sugars (g)	Sugar/Serving (%)
Tropicana Pure Premium^®^ Original (No Pulp)	236.6	22	9.7
Tropicana Pure Premium^®^ Original (No Pulp)	236.6	22	9.7
Minute Maid^®^ (made from concentrate)	236.6	24	10.6
Tropicana Light ‘N Healthy	236.6	10	4.4
Average	236.6 ± 0.0	19.5 ± 6.4	8.6 ± 0.0

*n* = 4. Data represents mean ± SD.

**Table 2:** Beverage nutritional facts for four commercially available apple juice brands.

Apple Juice Brands	Serving Size (mL)	Total Sugars (g)	Sugar/Serving (%)
Simply^®^ Fresh Apple Juice	236.6	25	11.0
Martinelli’s Apple Juice	295.7	39	13.8
Kroger Apple	236.6	27	11.9
Juicy Juice	236.6	27	11.9
Average	251.4 ± 29.6	29.5 ± 6.4	12.2 ± 0.0

*n* = 4. Data represents mean ± SD.

**Table 3:** Citric Acid reported for four commercially available orange juice brands by previous researchers.

Orange Juice Brands	Serving Size (mL)	Citric Acid (g/oz)	Citric Acid/Serving (%)
Tropicana Pure Premium^®^ Original (No Pulp)	236.6	0.277	0.98
Tropicana Pure Premium^®^ Original (No Pulp)	236.6	0.500	1.76
Minute Maid^®^ (made from concentrate)	236.6	0.240	0.85
Tropicana Light ‘N’ Healthy	236.6	0.494	1.74
Average	236.6 ± 0.0	377.7 ± 138.5	1.3 ± 0.0

*n* = 4. Data represents mean ± SD.

**Table 4:** PoTF (Power on Tag Forward) of three different tags on five different solutions and an empty PET control.

Beverage/Solution	Tag 1 PoTF (dBm)	Tag 2 PoTF (dBm)	Tag 3 PoTF (dBm)
Empty + PET	−20.78 ± 0.08 ^c^	−4.88 ± 0.11 ^b^	−1.43± 0.18 ^a^
DI Water + PET	8.37 ± 0.32 ^a^	4.19 ± 0.25 ^b^	−0.49 ± 0.05 ^c^
Orange Juice + PET	7.65 ± 0.52 ^a^	3.13 ± 0.15 ^b^	−1.57 ± 0.12 ^c^
1% Citric Acid + PET	9.67 ± 0.12 ^a^	3.38 ± 0.30 ^b^	−1.67 ± 0.05 ^c^
Apple Juice + PET	8.26 ± 0.08 ^a^	3.94 ± 0.22 ^b^	−2.01 ± 0.19 ^c^
12% Sucrose + PET	7.00 ± 0.34 ^a^	4.69 ± 0.12 ^b^	−1.43 ± 0.11 ^c^

Data are shown as mean value ± standard deviation (*n* = 30). Different letters (from a to c) within rows indicate significant difference (*p* < 0.05) between each tag.

**Table 5:** PoTR (Power on Tag Reverse) of three different tags on five different solutions and an empty PET control.

Beverage/Solution	Tag 1 PoTR (dBm)	Tag 2 PoTR (dBm)	Tag 3 PoTR (dBm)
Empty + PET	−23.65 ± 0.13 ^a^	−37.32 ± 0.43 ^b^	−38.16 ± 1.55 ^c^
DI Water + PET	−35.63 ± 0.32 ^a^	−46.74 ± 0.42 ^c^	−43.59 ± 0.23 ^b^
Orange Juice + PET	−35.56 ± 0.16 ^a^	−46.17 ± 0.33 ^c^	−43.01 ± 0.17 ^b^
1% Citric Acid + PET	−37.54 ± 0.32 ^a^	−46.17 ± 0.66 ^c^	−42.92 ± 0.18 ^b^
Apple Juice + PET	−35.62 ± 0.42 ^a^	−46.84 ± 0.41 ^c^	−42.04 ± 0.26 ^b^
12% Sucrose + PET	−35.34 ± 0.36 ^a^	−47.45 ± 0.32 ^c^	−42.84 ± 0.15 ^b^

Data are shown as mean value ± standard deviation (*n* = 30). Different letters (from a to c) within rows indicate significant difference (*p* < 0.05) between each tag.

**Table 6:** Best and worst angle and PoTF (Power on Tag Forward) from orientation sweep of three different tags on five different solutions and an empty control.

		Best Condition		Worst Condition		
		Angle (°)	PoTF (dBm)	Angle (°)	PoTF (dBm)	Dead Angles (°)
Tag 1	Empty	180	−21.34 ± 0.05	260	−7.24 ± 0.05	None
	DI Water	180	7.14 ± 0.04	80	9.38 ± 0.05	90–150, 210–270
	Orange Juice	50	7.08 ± 0.05	260	9.26 ± 0.05	110–250
	1% Citric Acid	60	7.28 ± 0.05	260	9.79 ± 0.06	110–250
	Apple Juice	310	7.64 ± 0.04	260	9.46 ± 0.05	100–250
	12% Sucrose	30	6.52 ± 0.00	230	9.64 ± 0.04	110–140
Tag 2	Empty	170	−5.44 ± 0.05	270	9.92 ± 0.00	70–90, 260
	DI Water	350	4.36 ± 0.05	300	9.86 ± 0.05	40–130, 220–290
	Orange Juice	350	3.00 ± 0.04	60	9.92 ± 0.00	70–130, 230–290
	1% Citric Acid	340	3.00 ± 0.05	290	9.75 ± 0.06	70–130, 220–280
	Apple Juice	350	3.92 ± 0.00	300	8.66 ± 0.09	50–140, 210–290
	12% Sucrose	350	4.40 ± 0.04	100	9.76 ± 0.05	110–150, 210–300
Tag 3	Empty	110	−4.68 ± 0.05	210	2.16 ± 0.00	None
	DI Water	80	−6.76 ± 0.04	170	9.58 ± 0.04	None
	Orange Juice	80	−6.50 ± 0.05	170	7.56 ± 0.00	None
	1% Citric Acid	80	−6.58 ± 0.05	170	7.28 ± 0.04	None
	Apple Juice	80	−7.80 ± 0.05	180	5.12 ± 0.05	None
	12% Sucrose	80	−6.2 ± 0.04	170	3.02 ± 0.05	None

*n* = 5. Data represents mean ± SD.

## Data Availability

The authors confirm that the data supporting the findings of this study are available within the article. All other relevant data supporting the findings of this study are available from the corresponding authors upon reasonable request.
